# Prevalence for delirium in stroke patients: A prospective controlled study

**DOI:** 10.1002/brb3.748

**Published:** 2017-06-23

**Authors:** Peter Nydahl, Gabriele Bartoszek, Andreas Binder, Laura Paschen, Nils G. Margraf, Karsten Witt, Andre Ewers

**Affiliations:** ^1^ Nursing research Campus Kiel University Hospital of Schleswig‐Holstein Kiel Germany; ^2^ Faculty of Health School of Nursing Science Witten/Herdecke University Witten Germany; ^3^ Department of Neurology University Medical Center Schleswig‐Holstein Christian‐Albrechts University Kiel Kiel Germany; ^4^ School of Medicine and Health Sciences ‐ European Medical School University Hospital of Neurology Medical Campus University of Oldenburg Germany; ^5^ Institute of Nursing Science and Practice Paracelsus Medical University Salzburg Austria

**Keywords:** Confusion Assessment Method, delirium, rehabilitation, stroke unit

## Abstract

**Background and Purpose:**

This study investigates the prevalence of delirium in acute stroke patients on a primary stroke unit (SU) analyzing associated risk factors and clinical outcomes.

**Method:**

Prospective, 4‐month observational study from 2015 to 2016 on patients aged ≥18 years with stroke at a German university hospital's SU. The presence of delirium as first outcome was rated at three times daily using the Confusion Assessment Method (CAM). Secondary outcome measures were duration of delirium, rehabilitation in SU, length of stay in SU and hospital, complications, and mortality. Significant risk factors were used to conduct a confounder‐matched case–control analysis.

**Results:**

309 patients were included. The overall prevalence of delirium was 10.7% (33 patients) mostly on the first and second hospital day. Duration of delirium on SU was in median 1.0 day (Interquartile range: 0.3–2 days). In 39.4% of patients delirium was present in a short time interval (≤8 hr) and in 24% of patients delirium was diagnosed during nightshifts exclusively. Significant risk factors for delirium were dementia, age ≥72 years, severe neurological disability on admission, and increased C‐reactive protein on admission. The case–control analysis showed that delirious patients had more complications and a trend toward a worse rehabilitation.

**Conclusions:**

These results underline the importance of delirium screening in stroke patients specifically during the night. Since even short delirious episodes are associated with more complications and increased disability, future studies are needed to find delirium prevention strategies.

## 1 INTRODUCTION

In Germany, 270,000 persons per year experience a stroke (Wiedmann et al., 2014). Common complications after stroke are dysphagia, aspiration pneumonia, falls, infections, depression, and delirium (Langhorne et al., 2000). Despite the high occurrence of delirium, a routine assessment of delirium for patients after stroke is not recommended (Norrving et al., 2015).

Delirium is defined as disorders in awareness and cognition (mainly attention and memory), develops within hours or days, cannot be explained by other cognitive disturbances as dementia and is a direct result of a physical disturbance or medication (American Psychiatric Association, 2013). Delirium can appear in hyper‐, hypoactive, or mixed forms, and substance withdrawal (Pandharipande, Jackson, & Ely, 2005). The etiology of delirium is complex, and current theories explain its development by interaction of hypoxia, inflammatory processes, disturbance of neurotransmitter, and the presence of internal or external risk factors (Riedel, Browne, & Silbert, 2014). The prevalence of delirium in patients after stroke is estimated to average 26% (Carin‐Levy, Mead, Nicol, Rush, & van Wijck, 2012). A delirium increases the risks for mortality, complications, longer length of hospital stay, and institutionalization (Shi, Presutti, Selchen, & Saposnik, 2012).

Till today, the prevalence of delirium on primary Stroke Units (SU) in Germany remains unknown. Hence, the purpose of this observational study was to evaluate the delirium prevalence in a German, national certified SU (Nabavi et al., 2015).

## 2 METHOD

We conducted a prospective, observational study on a SU over 4 months to assess prevalence of delirium in patients after stroke. Primary outcome was the presence of delirium. Stroke patients were examined for the presence of delirium three times a day. Diagnosis of delirium was assessed by using the Confusion Assessment Method (CAM) (Inouye et al., 1990), which is a screening algorithm, derived from the Diagnostic and Statistical Manual of mental Disorders, 4^th^ edition for the diagnosis of delirium. Secondary outcome parameters were duration of delirium, rehabilitation and first day of out‐of‐bed mobilization, number of delirium‐related pharmacological treatments, complications, length of stay on SU and in hospital, discharge destination, and mortality (all defined below).

### 2.1 Setting

The study was conducted in a primary, national certified SU (Nabavi et al., 2015). The SU has got a 24‐hr presence of a neurologist, interprofessional rounds twice a day, 2 weekly visits of pharmaceutics and antibiotica‐stewardship for patients with symptoms of infections. Nurse‐patient ratio is 1:4 in three shifts in 24 hr. 25% of 42 registered nurses joined a further education for specialized stroke care. Patients were cared by a comprehensive stroke treatment according with the German guidelines including regular assessment of the neurological status four times per day (06:00, 12:00, 18:00, and 22:00) by a neurologist with a continuous attendance on the SU; 2‐hourly observation of vital signs and neurological status by nurses and a permanent 24 hr bedside monitoring of vital parameters.

### 2.2 In‐ and exclusion criteria

Every patient, who was admitted on the SU, was screened for in‐ and exclusion criteria. Inclusion criteria were as follows: present ischemic or hemorrhagic stroke including transient ischemic attacks (TIA) (Wiedmann et al., 2014) and patients with cerebral venous sinus thrombosis. Exclusion criteria were (1) due to German law of data protection, no consent for research with patient's data by patients themselves or legal representatives; (2) patients with initial stroke‐like symptoms which could not be confirmed as stroke; (3) patients after neuroradiological interventions, because of a longer stay in hospital before the intervention; (4) patients who were admitted >24 hr on other units or hospitals; (5) patients with an age <18 years; (6) patients who were unable to be assessed for delirium; (7) other reasons, for example, foreign language. Criteria were confirmed by control of discharge information.

### 2.3 Risk factors and data collection

Based on a systematic review and post hoc analysis of risk factors in previous studies (Nydahl, Margraf, & Ewers, 2017), following patient factors were included: (1) socio‐demographical data: gender, age; (2) the presence of dementia and/or psychiatric disorders prior to admission, as reported by general practitioner; (3) C‐reactive protein (CRP) >0.4 mmol/L (4) admission in a 2‐ or 5‐bed room as environmental factor; and (5) status before admission: housing conditions and level of preexisting physical disability.

Level of disability was assessed using the modified Rankin Scale (mRS) (Banks & Marotta, 2007) that is a six‐item scale. Values from 0 to 2 are coded as nor or light disability and good outcome, values from 3 to 5 as severe disability and unwanted outcome. mRS was recorded before admission, during admission and at the point of discharge from SU. Rehabilitation on SU was calculated by difference between mRS at the time of discharge from SU and admission. Evaluation of National Institutes of Health Stroke Scale was not assessed routinely at all time points and could not be used for evaluation.

During the stay on the SU, the first day of mobilization was recorded as well as type and number of delirium related, pharmacological treatments were screened. Complications were assessed on a daily basis and categorized as: (1) falls; (2) urinary tract infection; (3) nosocomial pneumonia 48 hr after admission; (4) restraints of at least hands; and (5) unwanted removal of vascular‐, nasal‐, or bladder tubes.

### 2.4 Delirium assessment

Delirium was assessed using the CAM (Inouye et al., 1990). Assessment of CAM is based on four criteria: (1) acute onset and fluctuating course; (2) inattention; (3) disorganized thinking and/or (4) an altered level of consciousness (Inouye et al., 1990). Patients are assessed positive for delirium, if (1), (2) and either (3) and/or (4) are given, as described in detail on www.hospitalelderlifeprogram.org. The CAM has been validated for the assessment of delirium (Inouye et al., 1990), and can be used for patients after stroke (Dahl, Ronning, & Thommessen, 2010; Lees et al., 2013; McManus et al., 2009; Miu & Yeung, 2013) with a good sensitivity and specifity and has got a strong interrater reliability (Inouye et al., 1990). In case, patients had a severe aphasia and/or dementia and could not respond to simple questions, the presence of disorientated behavior and its fluctuation in 24 hr was rated as criteria for delirium (Gustafson, Eriksson, Sture, Bucht, & Gösta, 1991) and confirmed by families by asking them for new onset of such behavior. Assessment of delirium according to the CAM and its subtypes was conducted by nurses in each shift, three times a day covering a 24‐hr period (Lemiengre et al., 2006). Duration of delirium could be assessed for the stay on the SU. End of delirium was defined as 24 hr without any delirium‐positive assessment. In case, a patient was discharged from SU and not 24‐hr delirium‐free, the time of discharge was counted as end of delirium on SU.

### 2.5 Delirium management

Delirium screening was introduced in 2013. The interprofessional team was teached using a standardized script, bedside teachings, and case evaluations. Delirium‐Pocketcards and Posters were provided for clinicians, delirium‐information leaflets for families and patients. Families had no restrictions in visiting times. Delirium management included as first choice nonpharmacological interventions, including information, mobilization, reorientation, provision of glasses and/or hearing aids, sleep hygiene, and integration of families; and as second choice: pharmacological interventions.

### 2.6 Statistics

Nominal data are reported as frequency (*n*) and percentage (%). Metrical, normal distributed data were reported as mean and standard deviation, non‐normal distributed data as median and interquartile range (IQR). Calculated was length of stay by counting full days. Hypothesis was statistically proven by Fisher's Exact test for nominal data and Mann–Whitney U‐test for metrical data. To avoid misinterpretations by multiple testing, a sequential Bonferroni correction was used to correct for a two‐tailed α‐level of *p* = .05 by sequential division of number of factors included into the analysis(Bortz & Schuster, 2010). Multicollinearity was tested by Cramer's V and tolerated, if Variance of Inflation Factor <5 (Urban & Mayerl, 2008). Normal distribution of metrical data was tested by Shapiro–Wilk test (Bortz & Schuster, 2010). Due to the limited number of delirious patients, a logistic regression analysis could not be performed (Ottenbacher, Ottenbacher, Tooth, & Ostir, 2004). Hence, a matched case–control comparison was performed. Matching was conducted in a randomly chosen, 1:1 design without tolerance, using factors that were identified as significant after Bonferroni correction in previous bivariate analysis (Armenian, 2009). Group comparison between delirious cases and nondelirious controls were calculated using McNemar test, Yates correction, and Wilcoxon test, Odds ratios using Chi square (Bortz & Schuster, 2010). All calculations were done using spss 22 (IBM Corp. New York).

### 2.7 Ethical protocol approval

The study was approved by the ethic committee of Christian‐Albrechts‐University, Kiel. Due to the observational character of this study, the study was not registered.

## 3 RESULTS

**Figure 1:**
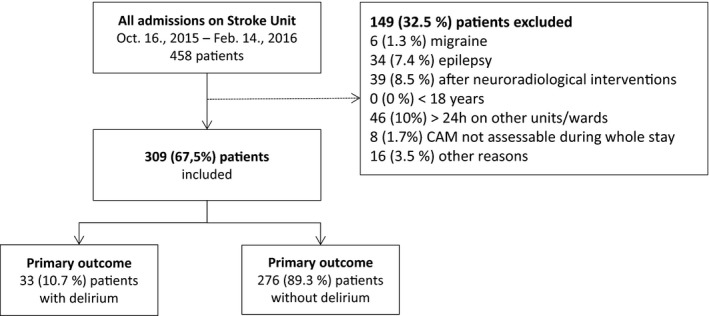
Recruitment of patients

The observational study covered 4 months from October 14th, 2015, till February 14th, 2016. Out of 464 admissions, 67.5% (*n* = 309) patients could be included (Figure 1).

### 3.1 Screening rate

The rate of delirium screenings in 309 patients was 84.3% (*n* = 1,747) of 2,071 possible delirium screenings. During the first week on SU, 40.6% (*n* = 685) assessments were conducted in morning shift, 28.5% (*n* = 482) in afternoon shift, and 30.8% (*n* = 520) in night shift. Delirium was not assessable in 5.5% (*n* = 17) of patients by several reasons, mostly during the first days. Out of these 17 patients, 70.6% (*n* = 12) became better and were assessable and all except one was free of delirium.

### 3.2 Delirium

Overall prevalence of delirium was 10.7% (*n* = 33) of patients. Delirium was assessed in 45.5% (*n* = 15) each on first and second day on SU, 9% (*n* = 3) of deliriums occurred during third or later days. Most delirium assessments identified a mixed delirium (57.7%, *n* = 41), followed by 19.7% (*n* = 14) in hyperactive form, 18.3% (*n* = 3) in hypoactive, and 4.2% (*n* = 3) in alcohol withdrawal form.

Duration of delirium was in median 1.0 days. (IQR: 0.3–2.0 days). Most delirious phases were less than 24 hr (45.5%, *n* = 15), 39.4% (*n* = 13) were delirious for only one assessment, hence, equal or less than 8 hr. 30.3% (*n* = 10) of delirious patients were discharged from SU, before they were 24‐hr delirium‐free. Delirium‐positive assessments were found in 5.4% (*n* = 37) morning shift, 6.8% (*n* = 33) in afternoon shift, and 8.6% (*n* = 45) in night shift. 24% (*n* = 8) of patients was delirious only during the night. Delirium‐related pharmacological treatment of delirium was administered to 69.7% (*n* = 23) of delirious patients. Most used medications for this reason were melperone (21.8%, *n* = 12), haloperidol (20%, *n* = 11), lorazepam (18.2%, *n* = 10), and others (40%, *n* = 22).

### 3.3 Comparison

Delirious and nondelirious patients were compared for risk factors. Significant risk factors for delirium after sequential Bonferroni correction were dementia (Odds Ratio (OR): 17.29, 95% Confidence Interval (95% CI): 6.745–44.322), severe neurological disability (mRS) on admission (OR: 6.791, 95% CI: 2.715–16.986), increased age ≥72 year. (median) (OR: 5.819, 95% CI: 1,992–17.002), increased CRP on admission (OR: 2.831, 95% CI: 1.338–5.989), and admission in 5‐bed room (OR: 0.216, 95% CI: 0.097–0.484). Multicollinearity was tolerable.

### 3.4 Case–Control

A randomized, 1:1 case–control design, matched for above listed significant risk factors could include 27 delirious and 27 nondelirious patients. Multicollinearity of risk factors was tolerable. Included patients (*n* = 54) differed significant from patients, who were not included in case–control design (*n* = 255): they were older (median: 80.5 year. (IQR: 75.0–87.2 year.) vs. 72.0 year. (60.0–81.0), *p* < .001), had a pronounced neurological deficit during admission (mRS: mean 3.7 (*SD* 1.2) vs. 2.3 (1.5), *p* < .001), had a longer stay on SU (median 3.0 days. (IQR: 2.7–5.0 days) vs. 3.0 days. (2.0–4.0 days), *p* = .006) and in hospital (8.0 days. (6.0–12.0 days) vs. 6.0 days. (4.0–10.0 days.), *p* = .008). The case–control analysis revealed that delirious patients showed significant more complications during their stay, but not a delayed mobilization, an increased length of stay on SU or in hospital nor higher mortality, compared to similar nondelirious patients. Compared to controls delirious patients showed a smaller success on rehabilitation with a difference of one point in the mRs on SU (uncorrected *p* = .017), which is clinically relevant. The difference is not significant after sequential Bonferroni correction, hence giving a trend to a decreased improvement in rehabilitation during their stay on SU (Table 1). The negative impact of delirium on rehabilitation is seen in patients with delirious episodes of 8 hr but is still evident in patients suffering from delirium less than 8 hr. (median: ‐0.54, IQR ‐3.0 ‐ 0.0).

**Table 1:** Case–control analysis for delirious and nondelirious patients

Variable	Total	Delirious	Nondelirious	*p* ‐value^a^
Number (%)	54 (100%)	27 (50%)	27 (50%)	
Risk factors				
Admission in 5‐bed room^b^	20 (37%)	8 (29.6%)	12 (44.4%)	.398
During stay on Stroke Unit				
Delirium‐related pharmacological treatment^b^	18 (33.3%)	17 (63%)	1 (3.7%)	.01^sign^
First mobilization during first two days^b^	40 (74.1%)	16 (59.3%)	24 (88.9%)	.042 ^ns^
Complications (at least one)^b^	19 (35.2%)	17 (62.9%)	2 (7.4%)	<.001^sign^
Outcome				
Rehabilitation on Stroke Unit (Difference mRS admission ‐ mRS discharge)^c^, ^d^	−0.67 (1.86)	−0.31 (1.8)	−1.31 (1.6)	.017 ^ns^
Length of stay on Stroke Unit (days)^e^	3 (2.7–5)	4 (3–6)	3 (2–4)	.094
Length of stay in hospital (days)^e^	8 (6–12)	8 (5–14)	7 (6–10)	.829
Mortality^b^	3 (5.5%)	2 (7.4)	1 (3.7)	1

a Sequential Bonferroni correction was conducted for each section (risk factors, during stay on Stroke Unit, Outcome).

^sign^, indicates a significant result after correction; ^ns^, indicates a nonsignificant result after correction for multiple comparisons.

b Number (%).

c Mean (standard deviation).

d Severe disability on admission (mRS 3‐5) had 22 patients in each group.

e Median (interquartile range).

## 4 DISCUSSION

In this prospective, observational study prevalence of delirium in more than 300 patients after acute stroke was nearly 11%. Screening of delirium was conducted three times a day and achieved a screening rate of more than 80%. Most delirious episodes were detected on first and second day after admission, during the night and lasted less than 1 day. In a case–control analysis, matched for dementia, severe neurological disability on admission, increased age ≥72 year., and increased CRP on admission, delirious patients had more complications but not a worse outcome except a tendency for a reduced rehabilitation improvement, compared to control patients without delirium.

Delirium is related to several risk factors as higher age, dementia, disability on admission, and increased CRP. Higher age was found as a risk factor in other studies, too (Miu & Yeung, 2013; Oldenbeuving et al., 2011) and may be explained by changed morphology of the aging brain (Oldenbeuving et al., 2011) or reduced perception (Dahl et al., 2010). Patients with dementia show a higher risk for delirium (Holtta et al., 2014), especially with infections (Simone & Tan, 2011). A severe disability on admission, assessed by the modified Rankin Scale, had also a higher risk for delirium, what can be explained by disturbed neurotransmitters, leading to delirium (Maldonado, 2008). An increased CRP is an indicator for infections, which are a trigger for delirium in general (Maldonado, 2008). After controlling for confounding risk factors (higher age, dementia, disability on admission, increased CRP), delirious patients in our cohort had not a worse outcome, compared to similar patients without delirium. This is in contrast to other studies (Caeiro, Ferro, Albuquerque, & Figueira, 2004; Gustafson et al., 1991; Mitasova et al., 2012). Severely disabled, delirious patients have more complications and might have a worse rehabilitation on SU, even in short episodes of delirium. Rehabilitation requires active involvement and patients’ alertness. Delirium disturbs these factors and may necessitate a prolonged rehabilitation. Beside, other hypothesis as more complicated infarction and reduced number of rehabilitation session due to delirium might also explain a reduced success in rehabilitation, and hence make it difficult to distinguish between cause and effect in this aspect. Due to the early onset of delirious episodes at first or second day, the effect of nonpharmacological and pharmacological prevention strategies on delirium prevalence, and hence rehabilitation, remains unproven. More research is needed to evaluate the impact of delirium on stroke rehabilitation.

Screening rate of delirium was above 80% and covered 24 hr. Only one other study used a 24‐hr screening, too (Gustafson et al., 1991), but found higher prevalence. Most positive screenings were during the night, hence screening rates of other studies, which used a once per day assessment, might have underestimated delirium prevalence (Caeiro et al., 2004; McManus et al., 2009; Miu & Yeung, 2013; Oldenbeuving et al., 2011). The assessment instrument CAM was used in other studies with patients after stroke, too with results of 10% (Dahl et al., 2010), 12% (Oldenbeuving et al., 2011), 27% (Miu & Yeung, 2013), and 28% (McManus et al., 2009). Delirium screened by nurses might be incorrect, especially in hypoactive delirium and dementia, leading to an underestimated prevalence (Lemiengre et al., 2006), contrary, a false‐positive delirium in one single shift would add another patient with delirium leading to an overestimated prevalence. Nevertheless, nursing staff was educated and cooperated with specialized physicians, leading to an overall correct screening. Due to the high rate of cases of delirium during the night, 24–hr screening is recommended for covering delirious episodes.

Prevalence of delirium is 10.7%, giving a lower prevalence than 26% in a recent meta‐analysis (Carin‐Levy et al., 2012). There are different hypothesis’ to explain this result. Improvements in stroke care over the last years may contribute to a low prevalence of delirium (Dahl et al., 2010). Especially close observation of neurological status and vital parameters (e.g., temperature together with fast escalating infection strategies) may reduce incidence of delirium as response to cerebral reaction to infection induced neurotoxic transmitters (Riedel et al., 2014). Furthermore, on the SU in our study delirium screening and evidence‐based delirium management is part of daily routine, including nonpharmacological interventions and early mobilization (Bernhardt et al., 2016). Other studies in the field did not reported delirium‐related structures and processes; hence a comparison is not feasible. Another influencing factor may be the inclusion of patients with TIA, who were included in other studies, too with a delirium prevalence of 10% (Dahl et al., 2010) and 48% (Gustafson et al., 1991). Patients with TIA are less impaired and this may reduce prevalence’ rates. Overall, we hypothesize that education of the personnel and delirium‐aware structures on a SU may have a positive impact on delirium prevalence, but a final proof in a delirium reducing setting of SU is still missing.

Duration of delirium was in median 1 day, 45% of delirious episodes lasted for 24 hr or less. In other studies, duration of delirium was in mean 4 days (Dostovic, Smajlovic, Sinanovic, & Vidovic, 2009; Mitasova et al., 2012) or 4.8 days (Oldenbeuving et al., 2011) and delirious episodes ≤24 hr were reported in 25% (Mitasova et al., 2012) to 46% (Sheng, Shen, Cordato, & Zhang, 2006). This phenomenon in patients with an acute stroke might be caused by cerebral dysregulation and associated with reduced perfusion, hypoxia, and disturbed neurotransmitters (Maldonado, 2008). Especially delirium during the night is discussed by disturbed melatonin circulation (Oldham, Lee, & Desan, 2016) and might explain the result of a delirium only during the night. Another hypothesis might be early infections during onset of stroke causing delirious episodes, for example, by aspiration and first cerebral responses, especially in patients with poststroke immunodepression (Famakin, 2014). Modern stroke care includes stabilization of circulation, narrow observation of vital signs in an appropriate nurse‐patient‐ratio, fast responses in terms of infections and implementation of nonpharmacological delirium prevention that may be an explanation for a short lasting delirium. Contrary, duration of delirium on SU can be influenced by length of stay on SU, too. In this study, 30% of delirious patients was discharged without being delirium‐free for 24 hr, to cover at least one following night without any delirium. Other authors defined the end of a delirium by one delirium‐free assessment (Oldenbeuving et al., 2011) or up to 48 hr without delirium (Mitasova et al., 2012). The question of the most appropriate definition of the end of delirium remains unanswered.

This study has different strengths and limits: A benefit of this study is the utilization of strict and explicit statistical methods. Data were collected over an extensive time period of 4 months on a primary stroke center with a high rate of accomplished delirium assessments. A limitation as a single center study is the missing option of a comparison of different SU settings in terms of delirium rates due to different SU concepts. Furthermore, the evaluation of the delirium duration is limited because some stroke patients were transferred to another ward before the end of delirium missing further follow‐up.

## 5 CONCLUSIONS

Delirium in stroke patients is frequent with a prevalence rate of 11%. An increased awareness toward these patients is required because of the significant more complications and their tendency for a decreased improvement in rehabilitation. A standardized screening of delirium, performed three times a day, is recommended to detect also short delirious episodes, especially during the night. Our use of stricter statistical methods helped to identify more reliable risk factors (mainly dementia, severe neurological disability as well as CRP on admission and age) and challenged results of other studies. Since even short delirious episodes are associated with more complications and increased disability, future studies are needed to find delirium prevention strategies.

## DISCLOSURES

Related to this study: none. Other disclosures: PN, AW, AB AE, and GB reported no conflicts of interest. KW reports personal fees from Medtronic, a travel grant from BIAL, grants from the Ministry of Research and the German Research Foundation, outside the submitted work.
